# ASL 4D MRA Intracranial Vessel Segmentation With Deep Learning U‐Nets

**DOI:** 10.1002/mrm.70173

**Published:** 2025-11-09

**Authors:** Sang Hun Chung, Zihan Wang, Tianrui Zhao, Zhitao Li, Chase S. Krumpelman, Sarah J. Moum, Sameer A. Ansari, Lirong Yan

**Affiliations:** ^1^ Department of Radiology Northwestern University Feinberg School of Medicine Chicago Illinois USA; ^2^ Department of Electrical and Computer Engineering McCormick School of Engineering, Northwestern University Evanston Illinois USA; ^3^ Department of Biomedical Engineering McCormick School of Engineering, Northwestern University Evanston Illinois USA; ^4^ Department of Medical Imaging Ann & Robert H. Lurie Children's Hospital of Chicago Chicago Illinois USA

**Keywords:** 4D MRA, arteriovenous malformation, ASL, deep learning, U‐net, vessel segmentation

## Abstract

**Purpose:**

To propose a spatio‐temporal U‐Net based network (4DST) that exploits both spatial and dynamic information while avoiding memory‐intensive 4D convolutional layers for ASL‐based non‐contrast enhanced 4‐dimensional MR angiography (4D MRA) vessel segmentation.

**Methods:**

Pulsed ASL‐based 4D MRA data were collected on 35 healthy volunteers and 5 arteriovenous malformation patients. Spatial only (2D, 3D) and spatio‐temporal U‐Net variations (including the proposed 4DST) were tested. Two recently developed methods, including feature‐based isolation forest and BRAVE‐Net, were used for comparison. Dice‐Sørensen coefficient (DSC), center‐line Dice (clDice), Hausdorff distance (HD), precision, accuracy, specificity, and sensitivity were calculated. Sensitivity was analyzed relative to SNR and arterial transit time (ATT) to explore detectability. From graph analysis, total vessel length, number of branches, and number of endpoints were reported.

**Results:**

4DST achieved the best DSC, clDice, and HD (0.876 ± 0.03, 0.865 ± 0.02, 6.241 ± 0.95, respectively). 4DST outperformed all other models across the SNR range of 1 to 10 and arterial transit time range of 500 to 800 ms in sensitivity. Last, the 4DST segmentations yielded total lengths and the number of branch splits that more closely matched the ground truths compared to the other models.

**Conclusion:**

The proposed 4DST network architecture offers an overall improvement in 4D MRA vessel segmentation performance over the compared methods and provides the framework for an end‐to‐end trainable model for spatio‐temporal datasets. Additionally, 4DST requires minimal pre/post‐processing steps, rendering it an attractive solution for pulsed ASL‐based 4D MRA vessel segmentation.

## 1 Introduction

Non‐contrast enhanced time‐resolved 4‐dimensional MR angiography (4D MRA) has demonstrated a promising non‐invasive MRA technique for diagnosing cerebrovascular diseases. By integrating arterial spin labeling (ASL) with cine acquisition, 4D MRA enables depicting the passage of blood flow through intracranial blood vessels at both high spatial and temporal resolutions. Recent advances in both image acquisition and reconstruction have further promoted 4D MRA as a reliable and time‐efficient technique [1, 2, 3, 4]. Compared to other imaging modalities, such as digital subtraction angiography (DSA), CT angiography (CTA), and contrast‐enhanced MRA (CE‐MRA), ASL‐based 4D MRA is a non‐invasive technique without the need for contrast injection.

Multiple studies have demonstrated the success of 4D MRA in clinical applications, such as the characterization of flow shunting in arteriovenous malformation [5] and imaging collateral circulations in steno‐occlusive diseases [6]. However, current applications of 4D MRA are limited to visual analysis of dynamic blood flow patterns. Segmentation of blood vessels from 4D MRA could provide further insight into vascular pathologies. Accurate vessel segmentation is an essential step for automatic morphological quantifications of the cerebral vasculature, which have been demonstrated to be useful imaging markers for cerebrovascular pathologies. For example, studies have shown that cerebral vessel density and tortuosity are associated with vascular pathologies, such as atherosclerosis [7], intracerebral hemorrhage [8], malignant tumors [9, 10], and white matter hyperintensities [11].

Driven by the recent advances in computational hardware, image processing, and artificial intelligence (AI) applications, cerebral vessel segmentation on MRA images has been significantly advanced in recent years. However, the majority of developments have been limited to the vessel segmentation of static MRA, for example, time‐of‐flight (TOF) [12, 13], which differs from 4D MRA in resolution and signal distribution. In recent years, some efforts have been made to segment ASL‐based 4D MRA using classical machine learning methods, such as a seed‐based algorithm [14] and an Isolation Forest algorithm [15]. In both studies, the inclusion of temporal dynamics has been shown to aid in segmentation. However, the need for extensive pre/post‐processing (such as skull stripping) and manual hyperparameter tuning could hamper their wide adoption in clinical use.

U‐Net [16] and its variations have found wide success in medical imaging applications through multiple imaging modalities and segmentation tasks [17, 18]. Recent work has demonstrated that the 3D U‐Net architecture provides better performance over other architectures in vessel segmentation [19, 20]. However, 3D U‐Net (and 2D) has primarily been applied to static image segmentation (including dynamic images that are first collapsed as a temporal maximum intensity projection [tMIP]), leaving a potential opportunity to improve vessel segmentation by leveraging the temporal dimension.

Inspired by the findings from Phellan et al. [14] and Liao et al. [15], the inclusion of temporal information could improve the performance of ASL‐based 4D MRA segmentation with deep learning (DL) U‐Net models. In this work, we introduce an altered U‐Net based deep learning architecture termed 4DST that incorporates both spatial and temporal information for 4D MRA vessel segmentation. We hypothesize that a U‐Net based architecture incorporating temporal information can improve ASL‐based 4D MRA vessel segmentation.

This study compared several U‐Net architectures (2D and 3D). For additional comparison, we also included BRAVE‐Net [20], a modern U‐Net improvement for volumetric vessel segmentation, and a non‐DL method [15] for 4D MRA vessel segmentation that combines temporal and spatial features using the Isolation Forest (IsoF) algorithm.

The performance of these methods was evaluated using quantitative metrics, including Dice‐Sorensen coefficient (DSC), center‐line Dice (clDice) [21], Hausdorff distance (HD) [22, 23], sensitivity, precision, accuracy, and specificity. Total vessel length from graph analysis [24, 25] was reported and compared with the ground truth. Segmentation sensitivity was explored as a function of signal‐to‐noise ratio (SNR) and arterial transit time (ATT). To test the generalizability of the proposed network, external validation was performed on five arteriovenous malformation (AVM) patients, and on the test‐set reconstructed with different numbers of MR acquisition spokes per frame.

## 2 Methods

This study was approved by the Northwestern University institutional review board. Written Informed consent was obtained for the data collection. Thirty‐five healthy volunteers participated in this study (34.1 ± 16.2 years old, 19 females). The collected data from healthy volunteers was split subject‐wise into training, validation, and testing sets in a ratio of 3:1:1. Additional data from five AVM patients (42.8 ± 27.6 years old, 1 female) were collected for external validation of the trained models.

### 2.1 MR Imaging

MRI scans were conducted on a Siemens MAGNETOM 3T Prisma system (Erlangen, Germany) using a 20‐channel head/neck coil. 4D MRA was acquired with a stack‐of‐stars (SOS) golden angle radial acquisition following a pulsed ASL preparation [26, 27]. The signal targeting with alternative radio frequency (STAR) [1] scheme was employed for the ASL preparation, in which the labeling plane was positioned inferior to the imaging volume. A pre‐saturation pulse was applied in the imaging slab right before the ASL preparation.

The imaging parameters of the 4D MRA sequence on all healthy volunteers were field of view (FOV) = 256 × 256 × 54 mm^3^; spatial resolution = 1 × 1 × 1.5 mm^3^; flip angle = 30°; TE/TR = 2.49/5 ms; 500 in‐plane radial spokes per partition (2.5 s acquisition window); 36 slices, scan time = 2 min 30 s. The 4D MRA sequence on AVM patients followed similar imaging parameters except for matrix size = 240 × 240 × 72 mm^3^; resolution = 1 × 1 × 1.3 mm^3^, 72 slices with acceleration factor = 2, 400 radial spokes per partition (2 s acquisition window) were collected, resulting in a scan time of 3 min 30 s.

A recently developed subspace low‐rank reconstruction [26] was applied to reconstruct 4D MRA images. 25 (healthy) and 20 (AVM) time frames with in‐plane 20 spokes per frame were constructed, resulting in a temporal resolution of 100 ms (training, validation, test sets). Additionally, 10 spokes (50 ms temporal resolution), and 40 spokes (200 ms temporal resolution) reconstructions from the test set were also generated for generalizability testing (external validation sets).

### 2.2 Data Preparation for Training, Validation, and Testing

The 4D MRA images were cropped (resulting in: 192 × 192 × 32 × 24 [XYZT]) to remove background and remain divisible by the network encoding down‐sampling steps. The temporal dimension (T) was smoothed with a moving average of window size = 3 to reduce outliers. The intensities were normalized per participant with *z*‐score normalization. tMIPs were performed before patch splitting for the spatial‐only models (2D, 3D, BRAVE‐Net). Out of the total 35 healthy participants, 7 participants were randomly selected and separated for future testing (20% of the total). The split between training and validation was performed after test‐set exclusion and patch generation. The split was done in a 3:1 (train: validation) ratio at the patch level.

For the 2D, 3DST, and 4DST models, the inputs were full slice‐based patches with matrix sizes of 192 × 192 [XY], 192 × 192 × 24 [XYT], and 192 × 192 × 3 × 24 [XYZT], respectively, which resulted in 28 × 32 = 896 patches for training and validation for the 2D and 3DST models. For the 4DST training and validation data sets, three adjacent slices of dimension [XYT] were concatenated in the Z dimension following the intact full volume slice ordering. The 3‐slice patches were extracted with a stride of one slice, resulting in 894 patches.

For the 3D and BRAVE‐Net models, the patches were volumetric patches with a size of 64 × 64 × 8 [XYZ]. In addition, context patches were extracted for BRAVE‐Net with a size of 128 × 128 × 16, following its original implementation. Accordingly, the resulting patch numbers for 3D and BRAVE‐Net were 4900 (stride 32 × 32 × 4) and 4116 (stride 10 × 10 × 8), respectively. For test inference, the seven test cases were prepared with no patch overlaps. Input dimensions, spatial versus temporal data summary, and number of patches are shown in Table 1.

**TABLE 1:** Model architectures and training summary.

Method	Spatial	Temporal	Input dimension	Trainable parameters	Train/val # patches	Batch size	Training time	Learning rate
IsoF	3D	✓	192 × 1922 × 322 × 24 [XYZT]	NA	NA	NA	NA	NA
2D_34	2D	**×**	1922 × 192 [XY]	7.80E+06	896	34	0.20 h	5e−4
3D_34	3D	**×**	642 × 642 × 8 [XYZ]	5.80E+06	4900	34	0.66 h	5e−4
BRAVE	3D	**×**	642 × 642 × 81282 × 1282 × 16 [XYZ]	1.01E+07	4116 center 4116 context	34	2.32 h	1e−4
2D_2	2D	**×**	1922 × 192 [XY]	7.90E+06	896	2	0.18 h	5e−4
3D_2	3D	**×**	642 × 642 × 8 [XYZ]	5.80E+06	4900	2	0.86 h	5e−4
3DST	2D	✓	1922 × 1922 × 24 [XYT]	1.10E+07	896	2	1.71 h	5e−4
4DST	3D	✓	1922 × 1922 × 32 × 24 [XYZT]	1.10E+07	894	2	5.84 h	5e−4

### 2.3 Data Preparation for External Validation

In the AVM set, only 400 radial spokes were collected due to scanning time constraints (resulting in 20 frames at a temporal resolution of 100 ms). Therefore, the temporal dimension was zero‐padded at the later frames to match the trained model's input. All other preparation steps were identical to the test set preparation.

To test the generalizability of different 4D MRA temporal resolutions, the seven healthy test cases were regenerated through reconstruction with 10 and 40 spokes per frame, resulting in temporal resolutions of 50 and 200 ms, respectively. Since reconstruction with different temporal resolutions resulted in different data lengths in the temporal dimension, the temporal dimension was either down‐sampled by averaging (in the 50 ms case) or up‐sampled by linear interpolation (in the 200 ms case) to match the original 100 ms temporal dimension size of the train data (*T* size = 24). For the spatial‐only models, the tMIP was calculated without the zero‐padding or temporal dimension resizing.

### 2.4 Ground Truth Generation

The ground truth vessel segmentations were generated by first smoothing the 4D MRA data in the time dimension with a moving average (window size 3, shift 1) and then cropping to a matrix size of 192 × 192 × 32 × 24. The temporally smoothed data were then used to calculate the level crossing count (LCC) in MATLAB (MATLAB version R2023b. Natick, Massachusetts: The MathWorks Inc.). The LCC is analogous to the more commonly used zero‐crossing count (ZCC), but the crossings are counted at the “level” line instead of at the zero line. The level was set manually to be over the noise level for each participant through visual inspection. The LCC was calculated for each voxel, resulting in a 3D matrix corresponding to the XYZ spatial dimension of the original image. The LCC matrix was then converted to a binary mask ((LCC > 1) = 1, (LCC ≤ 1) = 0).

While the purpose of the level and threshold was to remove background noise from the LZR calculation (acting as an intensity‐based threshold), the binarization step ((LCC > 1) = 1, (LCC ≤ 1) = 0) was to discern between blood flow from residual background signals.

Based upon the initial segmentation from LCC, the final binary masks were further refined with manual correction in 3D Slicer (https://www.slicer.org) [28]. The manual correction workflow consisted of first generating the temporal maximum intensity projection and loading the tMIP as a volume file in 3D Slicer. The LCC mask was loaded as a segmentation file. The tMIP was displayed in 3D using the volume rendering module, and the scalar opacity mapping was adjusted to improve vessel visibility. The LCC segmentation was also displayed in the 3D view as an overlay. The paint tool was used to draw missed vessels (distals and discontinuities), and the level tracing tool was used in the proximal greater vessels for consistent boundary annotation. Example images of the ground truth generation workflow are available in Figure S1. All ground truths were generated by a medical image researcher (6 years of experience).

### 2.5 Network Architectures

All tested models are based on the original U‐Net implementation by Ronneberger et al. [16], with slight variations. The encoding and decoding steps in all tested models were decreased from 4 to 3 steps due to our small matrix size in the temporal dimension. Instance normalization [29] was used after each convolution and before activation (convolution → instance normalization → activation). All convolution processes were done with zero padding by setting “padding = same” in TensorFlow Conv functions. The code was implemented in Python 3.10.14, TensorFlow 2.16.1, and Keras 3.1.1. The naming convention of the tested networks (2D, 3D, or 4D) was based on the network input dimensions. A summary of all models is shown in Table 1.

The 2D U‐Net has no variation from the original except for a decrease in encoding/decoding steps, instance normalization, and the final (kernel size = 1,1) convolution layer with sigmoid activation. The number of kernels was set to 64 (kernel size = 3,3 in all convolutions), starting in the first encoding stage and doubling each consecutive stage until the bottleneck. A similar progression was carried out on the decoding side, but with halving at each stage. The 3D spatial U‐Net consists of a similar architecture to the 2D U‐Net but with 3D convolution layers (kernel size = 3,3,3 in all convolutions) to account for the [XYZ] dimensions. Two 2D U‐Nets and two 3D U‐Nets were tested by varying training batch sizes (2 or 34), henceforth referred to as 2D_2, 2D_34, 3D_2, and 3D_34 (network diagrams shown in Figure S2).

**FIGURE 1:**
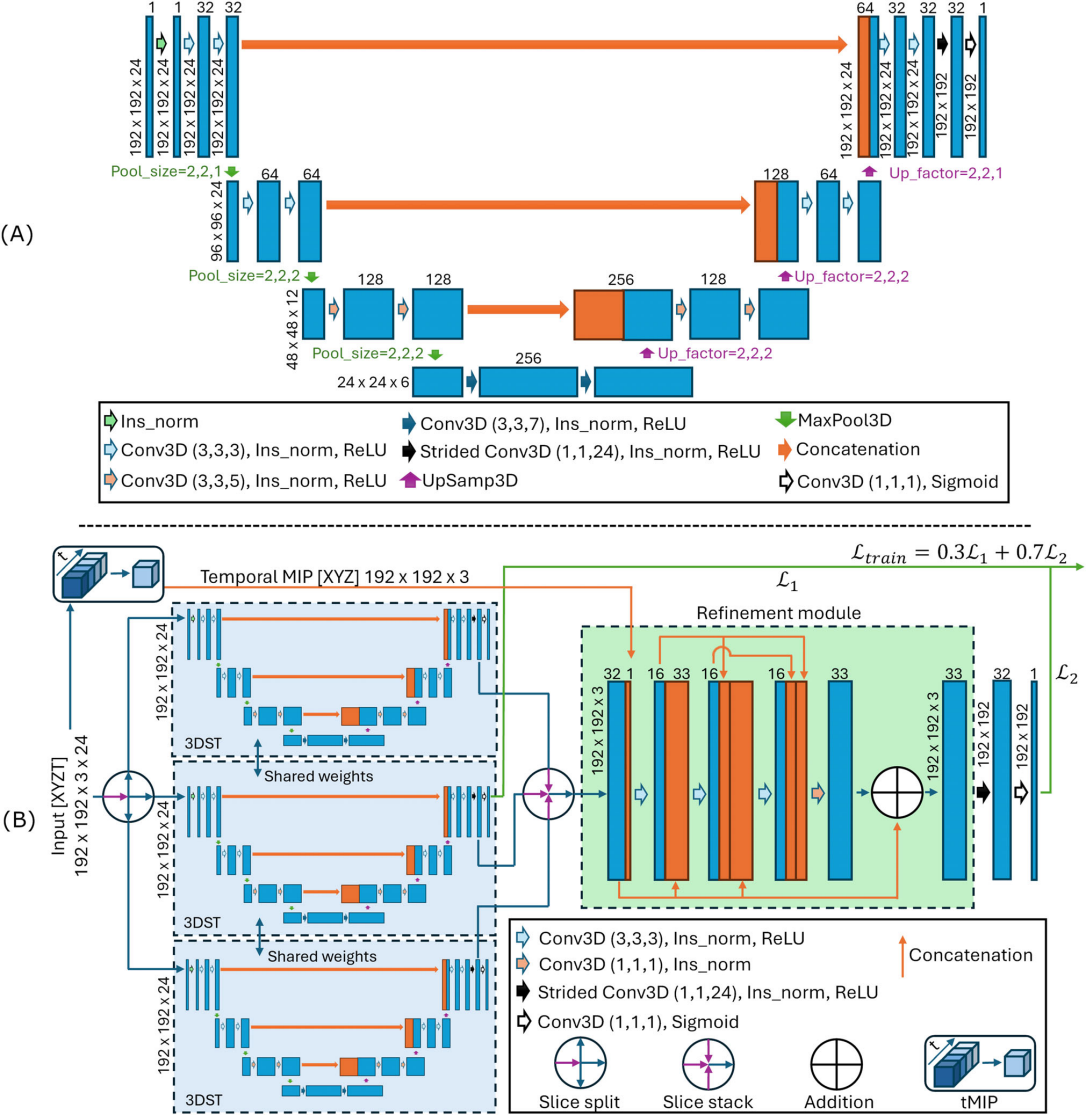
(A) 3DST U‐Net architecture showing feature sizes and number of channels. Down sampling pool sizes (green) and up‐sampling factors (purple) are shown at each stage. (B) 4DST U‐Net showing the parallel configured 3DST U‐Nets and the refinement module. The deep supervision pathway is shown with a green arrow. Concatenations are shown with orange arrows. Convolution kernel sizes are shown in the legends.

The input to the 3D spatio‐temporal (3DST) U‐Net takes full slice patches with a matrix size of 192 × 192 × 24 corresponding to the XYT spatial and temporal coordinates. The network output is a 2D segmented slice. The input is pre‐normalized with instance normalization (similar to BRAVE‐Net with batch normalization). The first maxpool and last upsamp are performed on the XY dimensions and not the time dimension. The convolutional kernel sizes in the time dimension were modified to be 3,3,5 in the encoding stages and 5,3,3 in the decoding stages. The kernel size at the bottleneck was set to 7 to increase temporal dynamic range. An extra unpadded convolution layer (kernel size = (1,1,24), strides = (1,1,1), instance normalization, ReLU), which collapses the features in the temporal dimension, was included before the final convolution layer (kernel size = (1,1,1), sigmoid) to output 2D segmented slices (Figure 1A). The 3DST design choices were guided experimentally with an ablation study (Table S1).

Last, the proposed 4DST model builds on top of 3DST. The input to the proposed 4D spatiotemporal (4DST) U‐Net was designed to accept 4D data corresponding to XYZT spatiotemporal coordinates (*Z* size = 3, corresponding to 3 adjacent slices). Three 3DST U‐Nets were arranged in parallel with shared weights as shown in Figure 1B. Each parallel arm is assigned an adjacent slice; the outputs of the arms are then concatenated and passed to the refinement module. The final two layers are a strided convolution layer (kernel size = (1,1,24), strides = (1,1,1), instance normalization, ReLU), which collapses the features in the temporal dimension, and the final convolution layer (kernel size = (1,1,1), sigmoid) to output 2D segmented slices. The design choice behind first performing segmentation on [XYT] data rather than [XYZ] was to have the convolutional operations of the 3DST applied along time. The loss function for the 4DST architecture is a weighted combination loss from the output of the middle arm (before the center 3DST [Conv kernel size = (1,1,1), sigmoid] layer) and the 4DST's final layer. The deep supervision loss path was used to allow the center arm 3DST to learn directly from ground truth, pushing training to be closer to a single 3DST and hopefully preserving the single 3DST performance on which the refinement module can improve. An example of similar use of deep supervision has been previously reported by Huang et al. [30].

The input of the 4DST refinement module is made by concatenating the features (before the Conv kernel size = (1,1,1), sigmoid layer) from the three parallel 3DSTs and the network's input tMIP. The tMIP concatenation acts as a skip connection. Four refinement modules were tested: a regular convolution (CNN), residual [31], dense [32], and ResDense [33] module. The residual architecture was included due to its ability to learn from reference, which has been shown to improve accuracy by He et al. [31]. For the dense architecture, Tong et al. [32] designed the architecture to propagate low‐level and high‐level features throughout the layers, and it was shown to alleviate the vanishing‐gradient problem. Resdense is a combination of the residual and dense design proposed by Zhang et al. [33]. All modules consisted of three convolution layers for fair comparison (Figure S3).

### 2.6 Isolated Forest and BRAVE‐Net

The isolation forest (IsoF) method was employed closely following the original paper [15] and the available source code. Briefly, Univariate Spline was used to interpolate the temporal frames to 100 frames. Nine temporal and eight spatial features were extracted on a voxel‐by‐voxel basis for classification. Isolation Forest parameters were kept at the default settings (100 decision trees, 256 voxel samples per tree, 1 feature with non‐replacement sampling). No postprocessing was applied for comparison fairness.

BRAVE‐Net was implemented closely following the original publication [20] with the published source code. There was no change in the network architecture. Other parameters included batch size = 34, learning rate = 1e−4, and DSC loss. A notable difference in our implementation was that all our extracted patches had vessels (due to cropping and larger voxel sizes); therefore, no sorting of vessel and non‐vessel patches was performed.

### 2.7 Network Training

The compared networks were trained with the Adam optimizer [34], a learning rate of 5e−4, and a batch size of 2, except for 2D_34, 3D_34, and BRAVE‐Net, which were trained with a batch size of 34 (following the original BRAVE‐Net implementation). The 2D_34 and 3D_34 models were included in our results for fair comparison with BRAVE‐Net. Batch sizes and learning rates for each model are shown in Table 1. Early stopping was utilized with a patience of 20 epochs. A learning rate reduction was applied with a factor of 0.1 and patience of 8 epochs. All training was done with an NVIDIA RTX A4500 and 12th Gen Intel(R) Core(TM) i7‐12700K 3.60 GHz.

The loss function used was a combination [35, 36, 37] of equally weighted DSC (Equation 1) and binary cross entropy (BCE) loss (Equation 2).

(1) $$\mathrm{DSC}=\frac{2\sum_{i}^{N}p_{i}g_{i}}{\sum_{i}^{N}p_{i}^{2}+\sum_{i}^{N}g_{i}^{2}}$$

(2) $$L_{\mathrm{BCE}}=-\frac{1}{N}\sum_{i}^{N}g_{i}\cdot \log\left(p_{i}\right)+\left(1-g_{i}\right)\cdot \log\left(1-p_{i}\right)$$

where *N* is the total number of voxels, *p* is the predicted output, and *g* is the ground truth. The combined loss equation is shown in Equation (3).

(3) $$L_{\text{train}}=\frac{1}{2}\left(L_{\mathrm{BCE}}\right)+\frac{1}{2}\left(1-\mathrm{DSC}\right)$$

All the tested models (except IsoF and BRAVE‐Net) used the loss function in Equation (3), including the 4DST network in both center arm and final layer. The final 4DST loss function was computed by a weighted sum between the center arm and the final layer.

### 2.8 Performance Metrics

The individually predicted slice segments were concatenated to reform the volumes in the test and external validation sets after inference. The DSC, clDice [21], HD [22, 23], sensitivity (or recall) (Equation 4), specificity (Equation 5), accuracy (Equation 6), and precision (Equation 7) were calculated per participant from the joined full volume segmentations.

(4) $$\text{sensitivity}=\frac{\mathrm{TP}}{\mathrm{TP}+\mathrm{FN}}$$

(5) $$\text{specificity}=\frac{\mathrm{TN}}{\mathrm{TN}+\mathrm{FP}}$$

(6) $$\text{accuracy}=\frac{\mathrm{TP}+\mathrm{TN}}{\mathrm{TN}+\mathrm{TP}+\mathrm{FN}+\mathrm{FP}}$$

(7) $$\text{precision}=\frac{\mathrm{TP}}{\mathrm{TP}+\mathrm{FP}}$$

where TP, TN, FP, and FN are true positives, true negatives, false positives, and false negatives, respectively. Sensitivity, specificity, accuracy, and precision were calculated with a tolerance of 1 voxel [38]. The decision to use a tolerance of 1 for the performance metrics was guided by previous studies [14, 39, 40] and to minimize partial volume annotation errors.

The total vessel length was calculated from network graph analysis. First, the FP voxels were removed from the predicted segments by multiplying with the binary ground truth to avoid erroneously inflated length values. Second, the predicted segments were thresholded with 0.5 to binarize the segments, skeletonized, and resized to isotropic resolution (by upsampling slices to match the in‐plane resolution). The skeletonization step for the network graph was computed with the MATLAB function bwskel(). Finally, vessel length, number of branches, and number of endpoints were calculated from graph analysis.

Sensitivity was also explored as a function of SNR and ATT. SNR was calculated for each voxel by dividing the tMIP signal by the standard deviation of a region of interest (ROI) outside the brain. ATT was calculated voxel‐wise by detecting the first time point above 3× the standard deviation of the background ROI [41, 42]. Both SNR and ATT maps were then masked with the vessel ground truth masks. The SNR and ATT maps were then binned (SNR bins [1–10, 11–20, ≥ 21] and ATT bins [100–400, 500–800, ≥ 900]) for analysis. All statistical tests in this study were conducted using a paired t‐test with a significance level of *p* < 0.05.

## 3 Results

### 3.1 4DST Design

The optimal weights used for the center arm and final layer loss in the 4DST network were determined empirically (Table 2A). The best DSC was achieved with a 30:70 ratio of center arm to final layer (DSC = 0.876 ± 0.03) and was used for the test and external validation results. 30:70 also resulted in the best clDice, HD, and accuracy. A ratio of 70:30 resulted in the highest specificity. Training with loss ratios of 0:100 and 100:0 did not converge. A ResDense‐based refinement module resulted in the best performance in most metrics (DSC, clDice, HD, sensitivity, accuracy) as shown in Table 2B. Therefore, a 4DST with ResDense refinement and loss weights of 30:70 was chosen for test and external validation metrics calculations.

**TABLE 2:** (A) 4DST ablation on deep supervision ratio, (B) 4DST ablation on refinement blocks, (C) model comparison performance metrics from test set (mean ± std.dev).

(A) 4DST DeepSup ratio (deep/end)	DSC	clDice	HD	Sensitivity	Specificity	Accuracy	Precision
0/100	0.015 ± 0.003	0.015 ± 0.003	57.7 ± 2.6	0.842 ± 0.01	0.48267 ± 0.01738	0.4963 ± 0.0184	0.061 ± 0.013
30/70	**0.876 ± 0.027**	**0.864 ± 0.022**	**6.2 ± 1.0**	*0.870* ± *0.05*	0.99985 ± 0.00005	**0.9984 ± 0.0005**	0.985 ± 0.005
50/50	0.864 ± 0.037	0.853 ± 0.030	6.5 ± 1.1	0.840 ± 0.07	*0.99986* ± *0.00005*	0.9981 ± 0.0007	*0.985* ± *0.006*
70/30	*0.870* ± *0.032*	*0.860* ± *0.025*	*6.4* ± *1.0*	0.856 ± 0.06	**0.99987 ± 0.00004**	*0.9983* ± *0.0006*	**0.986 ± 0.005**
100/0	0.029 ± 0.008	0.026 ± 0.006	63.5 ± 1.6	**0.921 ± 0.01**	0.57714 ± 0.03523	0.5926 ± 0.0357	0.096 ± 0.022

(B) 4DST Refinement	DSC	clDice	HD	Sensitivity	Specificity	Accuracy	Precision
Conv	0.864 ± 0.035	0.857 ± 0.028	6.7 ± 0.9	0.851 ± 0.08	*0.99987* ± *0.00005*	0.9982 ± 0.0008	*0.987* ± *0.006*
Residual	0.868 ± 0.036	0.859 ± 0.027	6.4 ± 1.0	*0.861* ± *0.07*	0.99985 ± 0.00005	*0.9983* ± *0.0007*	0.984 ± 0.007
Dense	*0.870* ± *0.033*	*0.862* ± *0.028*	*6.6* ± *1.0*	0.856 ± 0.07	**0.99988 ± 0.00004**	0.9983 ± 0.0007	**0.987 ± 0.004**
ResDense	**0.876 ± 0.027**	**0.864 ± 0.022**	**6.2 ± 1.0**	**0.870 ± 0.05**	0.99985 ± 0.00005	**0.9984 ± 0.0005**	0.985 ± 0.005

(C) Model	DSC	clDice	HD	Sensitivity	Specificity	Accuracy	Precision
IsoF	0.738 ± 0.049*	0.718 ± 0.072*	9.9 ± 2.0*	0.789 ± 0.05*	0.99909 ± 0.00076*	0.9966 ± 0.0011*	0.909 ± 0.082*
2D_34	0.841 ± 0.018*	0.836 ± 0.015*	7.2 ± 1.0*	0.847 ± 0.05*	0.99981 ± 0.00007*	0.9980 ± 0.0006*	0.980 ± 0.008*
3D_34	0.823 ± 0.022*	0.830 ± 0.017*	7.6 ± 1.1*	0.831 ± 0.06*	0.99979 ± 0.00006*	0.9979 ± 0.0006*	0.979 ± 0.007*
BRAVE	0.825 ± 0.033*	0.832 ± 0.025*	7.8 ± 1.1*	0.827 ± 0.08*	0.99975 ± 0.00012	0.9978 ± 0.0008*	0.976 ± 0.010
2D_2	0.847 ± 0.019*	0.847 ± 0.020*	7.3 ± 1.1*	**0.874 ± 0.06**	0.99983 ± 0.00006*	*0.9984* ± 0.0006*	0.983 ± 0.006*
3D_2	0.832 ± 0.027*	0.836 ± 0.020*	7.3 ± 0.9*	0.840 ± 0.07	0.99979 ± 0.00007*	0.9980 ± 0.0007*	0.979 ± 0.007*
3DST	*0.867* ± *0.034*	*0.856* ± *0.028*	*6.4* ± *0.9*	0.852 ± 0.07	**0.99986 ± 0.00003**	0.9982 ± 0.0007	*0.984* ± *0.005*
4DST	**0.876 ± 0.027**	**0.864 ± 0.022**	**6.2 ± 1.0**	*0.870* ± *0.05*	*0.99985* ± *0.00005*	**0.9984 ± 0.0005**	**0.985 ± 0.005**

*Note*: Best performing indicated in bold, second best italicized, * indicates *p* < 0.05 compared to 4DST.

### 3.2 Model Comparison

**FIGURE 2:**
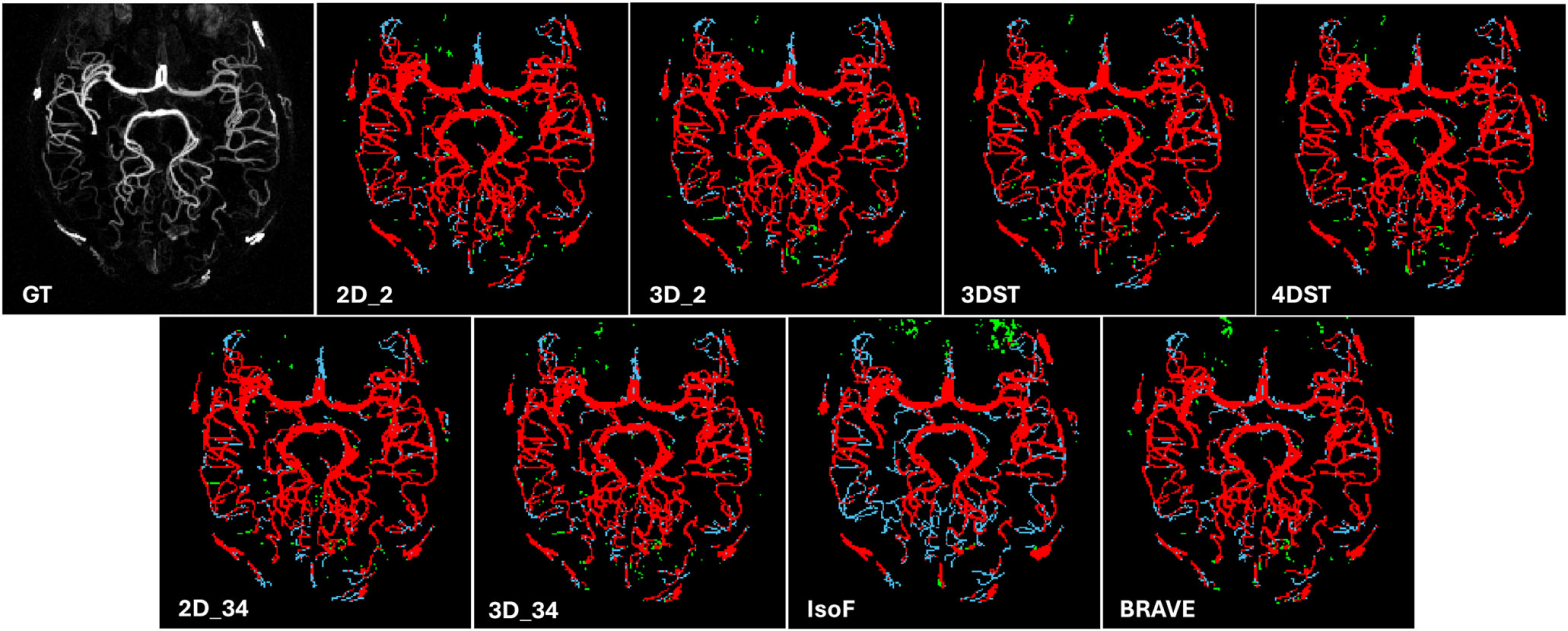
Example of test inference result shown as MIP images. Color coded to show TP (red), FN (light blue), and FP (green). Top left shows the input 4DMRA MIP image in grayscale.

The performance metrics obtained from the test set are listed in Table 2C. The 4DST and 3DST U‐Nets outperformed all other models in most metrics, indicating that the inclusion of temporal information on U‐Nets has a favorable impact on vessel segmentation. 4DST in particular had the highest DSC, clDice, HD, accuracy, and precision. Contrary to modern trends favoring 3D U‐Nets, the 2D U‐Nets outperformed their same batch size 3D counterparts and BRAVE‐Net in most metrics. BRAVE‐Net outperformed 3D_34 in DSC and clDice, but not in HD. Both 2D U‐Nets (2D_34, 2D‐2) outperformed BRAVE‐Net in DSC, clDice, and HD. Of note is that 2D_2 had the best sensitivity and second‐best accuracy among all tested models. Figure 2 shows MIP maps of TP, FP, and FN images from a representative participant. Training curves are shown in Figure S4.

### 3.3 Skeleton and Graph Analysis

**FIGURE 3:**
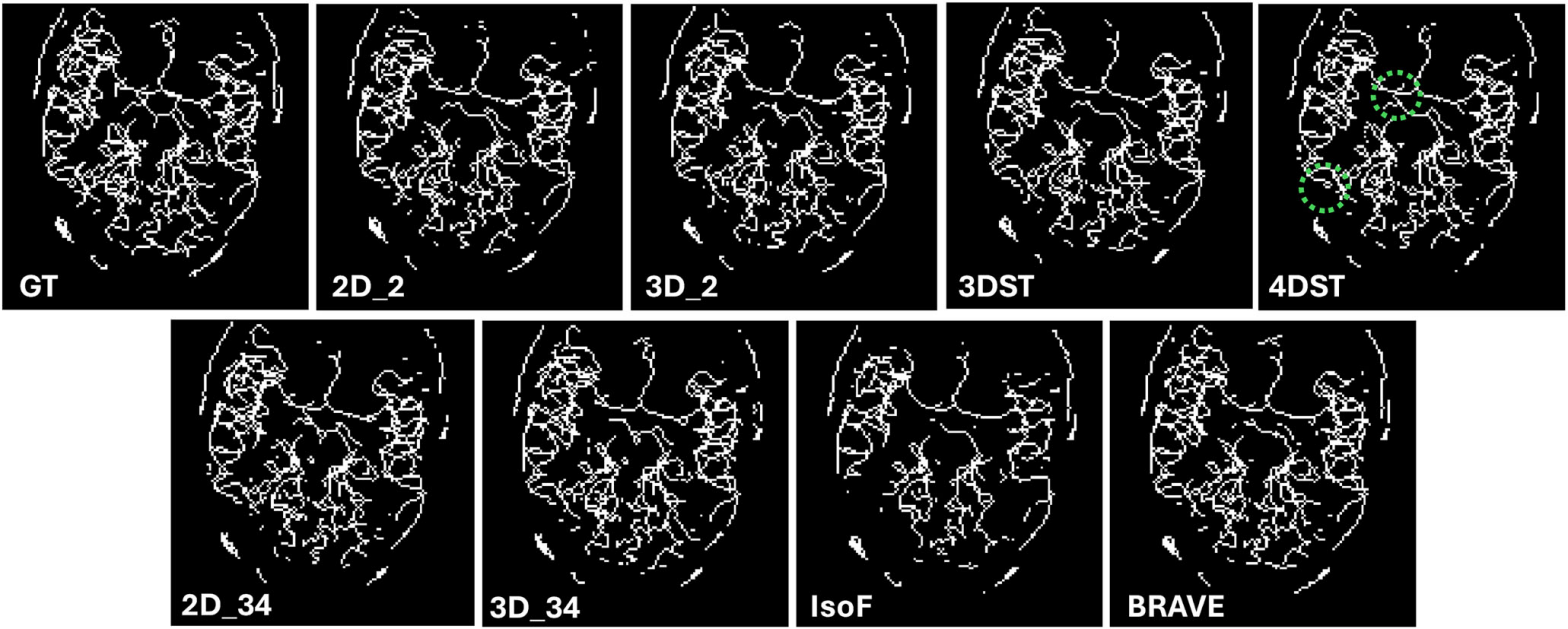
MIP images examples from the test‐set. The ground truth (GT) is included for reference. Examples of 4DST outperformance are highlighted in dashed circles.

The skeletonization results from a representative case are shown in Figure 3 along with the skeletonized ground truth. Table 3 shows the resulting metrics from the skeletons as an average difference to ground truths. All tested methods yielded visibly lower total vessel lengths when compared to the ground truth (average differences to ground truth ranged from 742.4 to 1828.3 mm). The mean total length and number of branch splits values of 4DST were the closest to the ground truth. 3D_2 had the most similar number of endpoints to the ground truth. However, based on the lower performance on total length and branch splits, this could be an indication of a higher prevalence of vessel discontinuities.

**TABLE 3:** Graph analysis metrics showing difference to ground truth (Δ = ground truth–model) (mean ± std.dev).

Model	ΔTotal length (mm)	ΔBranch splits	ΔEndpoints
IsoF	1828.3 ± 514.1*	47.1 ± 13.5*	38.7 ± 27.2*
2D_34	924.4 ± 216.3*	26.1 ± 13.9	*−2.1* ± *41.6*
3D_34	795.5 ± 179.8	*17.4* ± *9.9*	−26.6 ± 40.8
BRAVE	861.6 ± 237.4	27.0 ± 14.0*	−2.9 ± 26.2
2D_2	823.6 ± 224.4	20.9 ± 11.2	2.7 ± 29.6
3D_2	751.3 ± 223.3	22.7 ± 7.8*	0.3 ± 21.8*
3DST	*772.4* ± *243.9*	23.1 ± 13.6	−18.7 ± 27.3
4DST	**742.3 ± 251.6**	**16.3 ± 10.4**	−9.4 ± 25.6

Ground Truth	Total length (mm)	Branch splits	Endpoints
	6216.1 ± 1032.2	141.7 ± 32.5	236.5 ± 56.1

*Note*: Best performing indicated in bold, second best italicized, * indicates *p* < 0.05 compared to 4DST. Absolute ground truth values reported in the last row for reference.

### 3.4 Sensitivity Versus ATT and SNR

**FIGURE 4:**
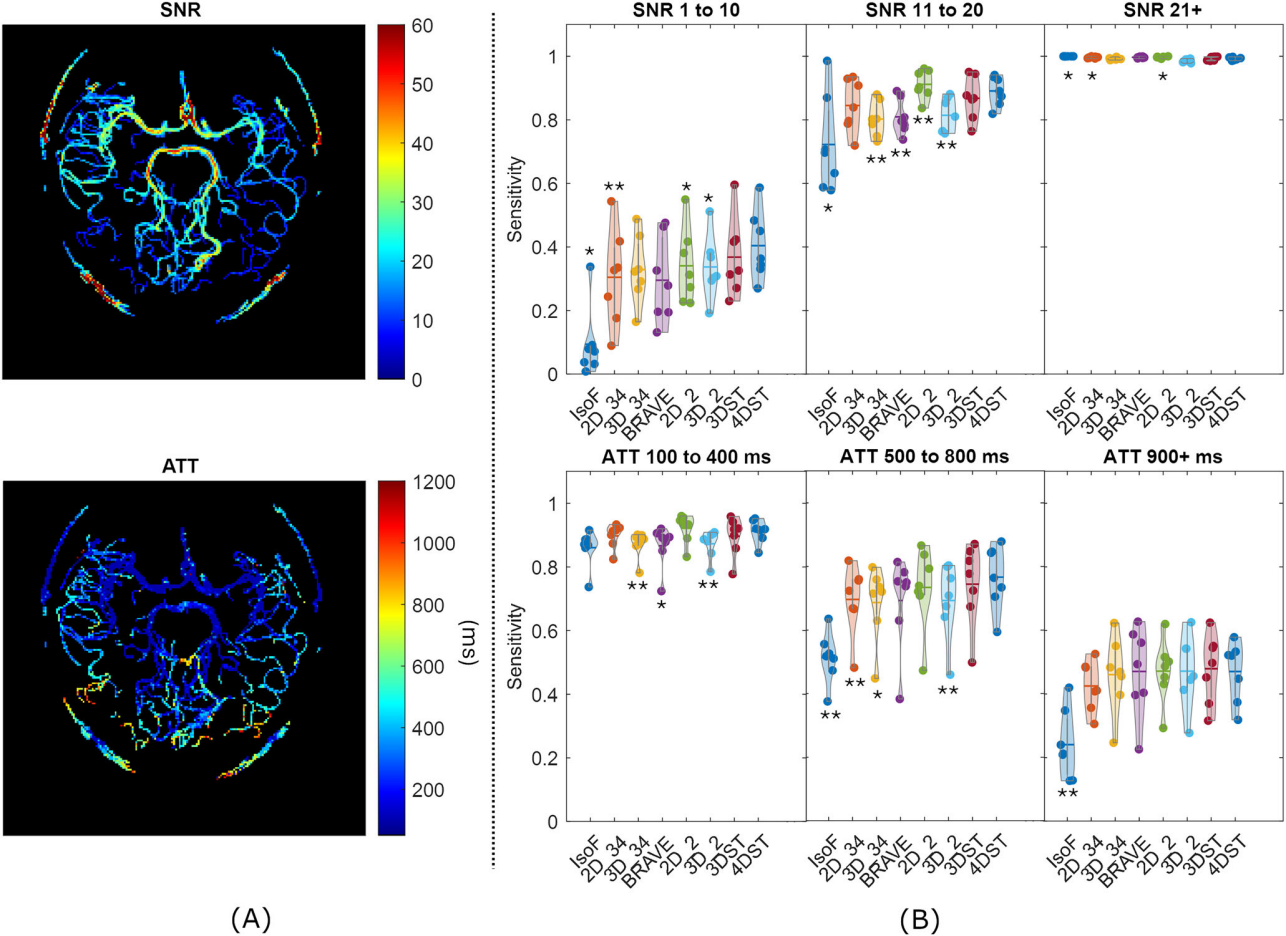
(A) MIP heatmaps of signal to noise ratio (SNR) and arterial transit time (ATT), (B) test‐set sensitivities at SNR and ATT (ms) ranges. Statistical significance is shown with *p < 0.05 and **p < 0.01 of each model with 4DST (paired t‐test).

As expected from ASL‐based 4D MRA, distal vessels have longer ATT and lower SNR due to blood T1 decay and increased partial volume effect (Figure 4A). Predictive sensitivity decreased as ATT increased. Likewise, predictive sensitivity increased as SNR increased for all models (Figure 4B). All models performed well in the SNR range of 21 and above (mean sensitivity > 0.992), and moderately well at the ATT range of 100 to 400 ms (mean sensitivity > 0.861). 4DST achieved the highest sensitivity among the models in the SNR range of 1 to 10, although there was no statistical difference between 3DST and 4DST. 4DST was significantly better than 2D_2 (third best performer after 3DST) in the SNR range of 1 to 10 (*p* = 0.041). 4DST outperformedall other models in the ATT range of 500 to 800 ms, but there was no statistical difference from 3DST, 2D_2, and BRAVE‐Net. Surprisingly, 2D_2 had the best sensitivity in the SNR range of 11–20 with statistical significance to 4DST (*p* = 0.001).

### 3.5 External Validation

**FIGURE 5:**
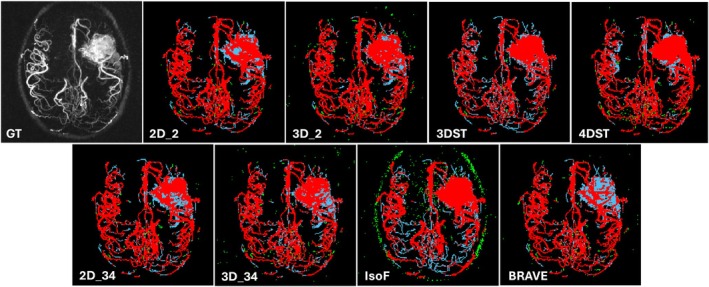
Example MIP AVM patient inference results. Color coded to show TP (red), FN (light blue), and FP (green). Top left shows the input 4DMRA tMIP image in grayscale.

The external validation results for 4DST are shown in Table 4. The full results of all models are provided in Table S2. From the five AVM patient data sets, 4DST exhibited slightly decreased performance when testing on the whole intracranial volume. The decrease in performance was less pronounced when performance was tested in the cropped AVM lesions, indicating that the decrease in 4DST performance is not driven by the AVM lesion but likely due to the missing last data points. Figure 5 shows example segmentation results for all models.

**TABLE 4:** 4DST external validation results (mean ± st.dev).

Metric	AVM whole volume	AVM cropped nidus	Healthy 10‐spoke	Healthy 40‐spoke
DSC	0.792 ± 0.008	0.852 ± 0.016	0.868 ± 0.027	0.856 ± 0.024
clDice	0.790 ± 0.01	0.828 ± 0.07	0.856 ± 0.022	0.854 ± 0.019
HD	10.1 ± 1.6	7.6 ± 3.3	6.4 ± 0.9	6.6 ± 0.8
Sensitivity	0.871 ± 0.038	0.902 ± 0.040	0.862 ± 0.041	0.856 ± 0.047
Specificity	0.99961 ± 0.00011	0.99924 ± 0.00028	0.99988 ± 0.00005	0.99976 ± 0.00008
Accuracy	0.9982 ± 0.0005	0.9915 ± 0.0055	0.9984 ± 0.0005	0.9981 ± 0.0006
Precision	0.962 ± 0.005	0.980 ± 0.022	0.988 ± 0.003	0.974 ± 0.010

**FIGURE 6:**
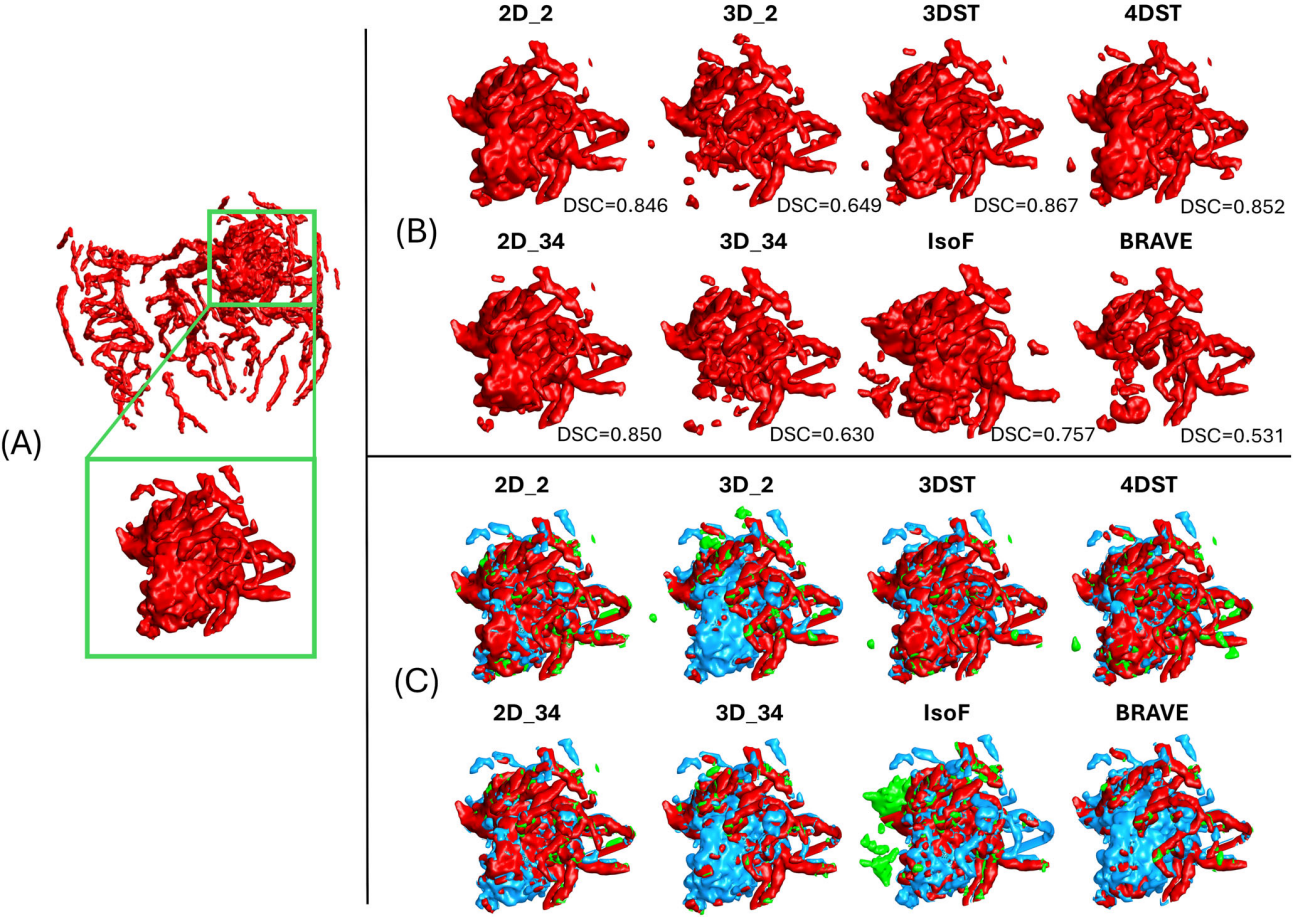
(A) Example ground truth of AVM patient and zoomed AVM lesion, (B) render of inference segmentation results with average DSC, and (C) color coded renders to show TP (red), FN (light blue), and FP (green).

Looking at the cropped AVM lesions (Figure 6), there is a noticeable trend between the models that infer slice by slice to the models that infer on 3D spatial volumes (i.e., learn from through‐slice information). The models that learned through slice information (3D_2, 3D_34, and BRAVE‐Net) exhibited a marked decrease in AVM lesion detection. A closer look at the rendered AVM lesion segmentation results (Figure 6B) indicates that those models are biased towards vessel‐like structures, resulting in missed dense areas of the lesion. In contrast, the decrease in performance of 4DST does not seem to be from missed dense areas (Figure 6C) but from increased false positives. This is further supported by comparing the sensitivity of 4DST to 3DST (0.901 ± 0.040 and 0.841 ± 0.041, respectively).

Analyzing the 10 and 40 spoke reconstructed test sets, the 4DST inference resulted in a small decrease in performance (20‐spoke test set DSC = 0.876 ± 0.027, 10‐spoke DSC = 0.868 ± 0.027,40‐spoke DSC = 0.856 ± 0.024). The 10‐spoke performed better than the 40‐spoke. 4DST performed better than all other models in DSC, clDice, and HD.

## 4 Discussion

4D MRA provides both spatial information of brain vessel structure as well as temporal information of arterial flow dynamics. Studies on the effect of temporal information inclusion in DL for vessel segmentation remain scarce. Our results showed that the addition of temporal information aided in vessel segmentation, achieving higher DSC as evidenced by the 3DST and 4DST scores. This finding is consistent with previous findings on ASL‐based 4D MRA segmentation with non‐DL methods [14, 15]. Of note is that the segmentation predictions in this paper were performed with minimal preprocessing steps.

4DST and 3DST achieved the best and second‐best DSC, clDice, HD, and precision, respectively. For 4DST, the inclusion of multiple slices (i.e., third spatial dimension) and the refinement module proved advantageous, yielding better performance in allmetrics on the test set over 3DST. 2D consistently outperformed 3D. This might be due to voxel anisotropy, which may degrade the performance of U‐Nets with 3D spatial convolutions as discussed in previous studies [43, 44, 45].

All tested DL models performed better than the Isolation Forest method. When tested with our data, the Isolation Forest achieved DSC of 0.738 ± 0.05, and clDice of 0.718 ± 0.07, all of which are lower than those reported in the original paper (e.g., DSC_small vessel_ = 0.871 ± 0.073, DSC_medium vessels_ = 0.912 ± 0.034) [15]. This discrepancy is likely due to the differences in the 4D MRA reconstruction and its resulting noise profile. Some temporal features used in the original publication failed to generate meaningful background‐vessel separation when applied to our data. Notably, the temporal number of peaks (numP) feature, which was reported to be the most important feature in the original work, failed to discern vessels. The employed low‐rank reconstruction in our study effectively flattens noise, making temporal features such as numP less effective. The low comparable performance of IsoF is likely a function of inadequate features specific to our data set and not the isolation forest algorithm.

Using the full 4D matrix [192 × 192 × 32 × 24] with 4D convolutions would require ˜62 GB of training memory, assuming parameter type of float32, batch = 2, kernel sizes = [3 × 3 × 3 × 3], and three encoder/decoder steps with channel sizes = [32 64 128 256 128 64 32] (each stage). Employing smaller patch inputs of size [192 × 192 × 8 × 24] (8 slices) would require ˜15.7 GB of memory, limiting training to modern high‐end GPUs. Further decreasing the patch size to [64 × 64 × 8 × 24] would result in ˜2 GB of required memory. Using smaller patches would be feasible in most computer setups at the sacrifice of the in‐slice context information. True 4D convolution U‐Nets for segmentation have been attempted in cardiac computer tomography (CT) with sparce annotations [46], but most opt to employ hybrid approaches. At the time of writing this study, the potential advantages and drawbacks of true 4D convolution U‐Net on ASL‐based 4D MRA remain understudied.

This study has limitations. Limited datasets were available in the study. Although the lack of data were somehow ameliorated by splitting the 3D volumes by slice and smaller 3D patches, training with more data and the incorporation of data augmentation techniques might yield better generalizable models and improve performance. All training data used in this paper came from healthy volunteers, which likely affected the segmentation performance of the AVM set. All the results presented in this study are specific to the mentioned mri protocol (pulsed ASL, SOS golden angle readout, and subspace low‐rank reconstruction) and require validation for different ASL labeling schemes, readout, and reconstruction methods. The generalizability to different pulsed ASL‐based 4D MRA temporal resolutions is also limited due to our test data generating from the same acquisition but with different reconstructions. A more comprehensive generalization test would require separate 4D MRA acquisition and reconstruction at the target temporal resolution. Generalization to other cerebrovascular diseases, such as atherosclerosis, aneurysms, and moyamoya, was not tested and requires further validation. Likewise, the generalization to other imaging protocols (including imaging modalities, hardware vendors, coils, and acquisition parameters) was not tested and require further validation.

Another limitation in this study is that skeletonization might have created errors due to missing voxels inside thicker vessels, tightly bending vessels, and irregular edge vessels. Lastly, our proposed method was not validated on its ability to provide clinically relevant metrics. Whether the current performance is sufficient for clinically relevant analysis of blood flow remains an open question.

For future work, while the segmentation performance metrics reported in this study achieved high results, the visibly lower total lengths from graph analysis compared to the ground truth indicate that there is still much room for improvement. One possible way to tackle this problem would be to use specialized loss functions such as clDice or a skeleton length maximizing loss. However, using specialized loss functions may over‐constraint training to be biased to tubelike structures leading to poor generalizability on vessel abnormalities. Inclusion of AVM data in the training stage might lead to better AVM lesion segmentation. The low segmentation performance at higher ATTs (≥ 900 ms) is another area of improvement requiring further attention. The higher ATT zones are distal vessels, which also suffer from low SNR due to blood T1 relaxation common in spatially‐selective ASL. Improvements in labeling methodology, less prone to ASL signal decay could improve segmentation performance. Additionally, the effects of SNR and ATT on sensitivity could be further analyzed by performing regression analysis to study their independent effects.

Different network architectures, such as processing tMIP [XYZ] first, then refining with the temporal data, might be an alternative to our work and requires further research. Another aspect not tested in this study is that the principal components from the low‐rank reconstruction could be leveraged for better segmentation performance. Last, the base 3DST, which acts as a modular branch in the 4DST, could be further improved. For example, using residual, dense, or ResDense blocks might yield better performance and merit further research.

## 5 Conclusion

This study demonstrated the feasibility of pulsed ASL‐based 4D MRA vessel segmentation with an end‐to‐end trainable U‐Net based architecture that incorporates 3D [XYZ] spatial and temporal information without memory‐intensive 4D convolutions. Our results indicate that the inclusion of temporal information resulted in higher DSC, clDice, and HD. The proposed refinement architecture further increased performance over the spatio‐temporal 3DST, supporting its inclusion. The proposed 4DST yielded high DSC scores without sophisticated pre‐processing, such as prior skull removal or whole brain segmentation. This work also showed how network design can affect vessel segmentation generalizability to abnormally shaped vessels.

## Supporting information


**Figure S1:** Example images from stages in the Ground truth generation pipeline. LCC = level cross count.
**Figure S2:** (A) 2D U‐Net architecture (B) 3D U‐Net architecture. Convolution kernel sizes are shown in the legend.
**Figure S3:** Tested refinement modules for 4DST. The last 2 layers, strided convolution and convolution with sigmoid activation are present in all modules. Convolution kernel sizes are shown in the legend.
**Figure S4:** Training curves of compared models, 2D_34 and 3d_34 correspond to models trained with batch size = 34, 2D_2 and 3D_2 correspond to models trained with batch size = 2. BRAVE‐Net was trained with batch size = 34. 3DST and 4DST were trained with batch size = 2.
**Table S1:** 3DST ablation, kernel sizes and pre‐normalization layer (instance normalization) (mean ± std.dev).
**Table S2:** External validation DSC metrics from AVM data set (whole volume and cropped AVM lesion), 10 and 40 spoke 4DMRA test‐set (50 and 200 ms temporal resolution respectively), and slice downsampled test‐set (mean ± std.dev).

## Data Availability

The source code and trained weights of the proposed network are provided at https://github.com/NITTLab001/4DMRA_4DST_segmentation.
