# Supplementary material for: ASL 4D MRA Intracranial Vessel Segmentation With Deep Learning U‐Nets
Source: Magn Reson Med. 2025 Nov 9;95(4):2384–96. doi: 10.1002/mrm.70173 (PMC12850589; doi:10.1002/mrm.70173)
Supplement: Supplementary file 1 — Figure S1: Example images from stages in the Ground truth generation pipeline. LCC = level cross count. Figure S2: (A) 2D U‐Net architecture (B) 3D U‐Net architecture. Convolution kernel sizes are shown in the legend. Figure S3: Tested refinement modules for 4DST. The last 2 layers, strided convolution and convolution with sigmoid activation are present in all modules. Convolution kernel sizes are shown in the legend. Figure S4: Training curves of compared models, 2D_34 and 3d_34 correspond to models trained with batch size = 34, 2D_2 and 3D_2 correspond to models trained with batch size = 2. BRAVE‐Net was trained with batch size = 34. 3DST and 4DST were trained with batch size = 2. Table S1: 3DST ablation, kernel sizes and pre‐normalization layer (instance normalization) (mean ± std.dev). Table S2: External validation DSC metrics from AVM data set (whole volume and cropped AVM lesion), 10 and 40 spoke 4DMRA test‐set (50 and 200 ms temporal resolution respectively), and slice downsampled test‐set (mean ± std.dev). [file MRM-95-2384-s001.pdf]

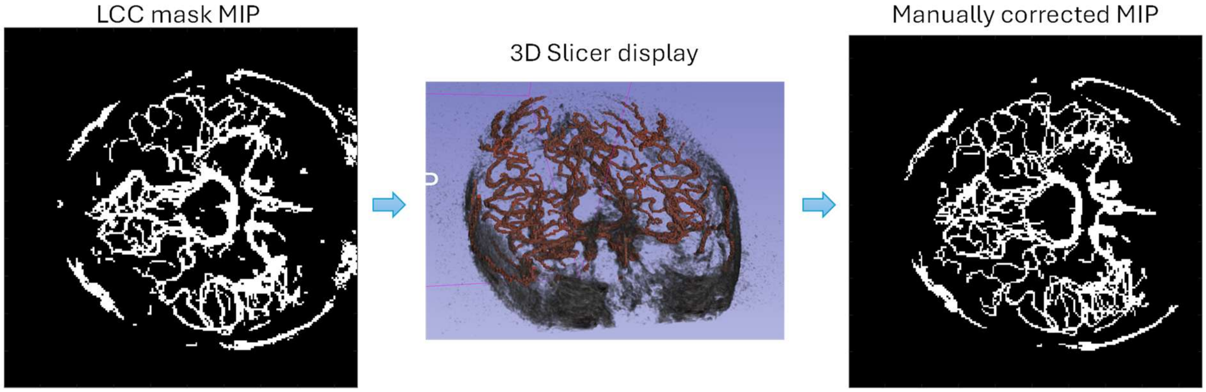

Figure S1: Example images from stages in the Ground truth generation pipeline. LCC = level cross count.

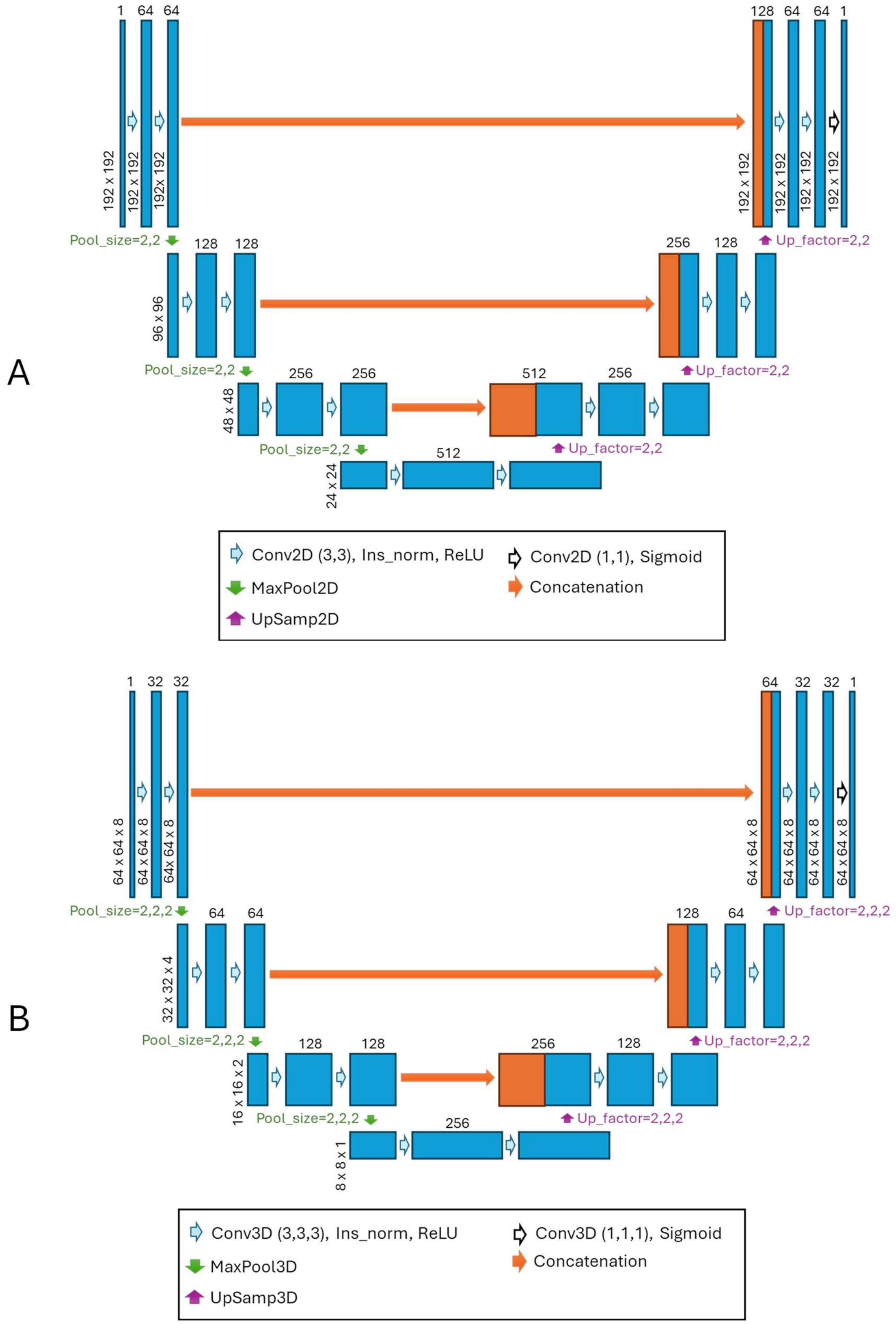

Figure S2: (A) 2D U-Net architecture (B) 3D U-Net architecture. Convolution kernel sizes are shown in the legend

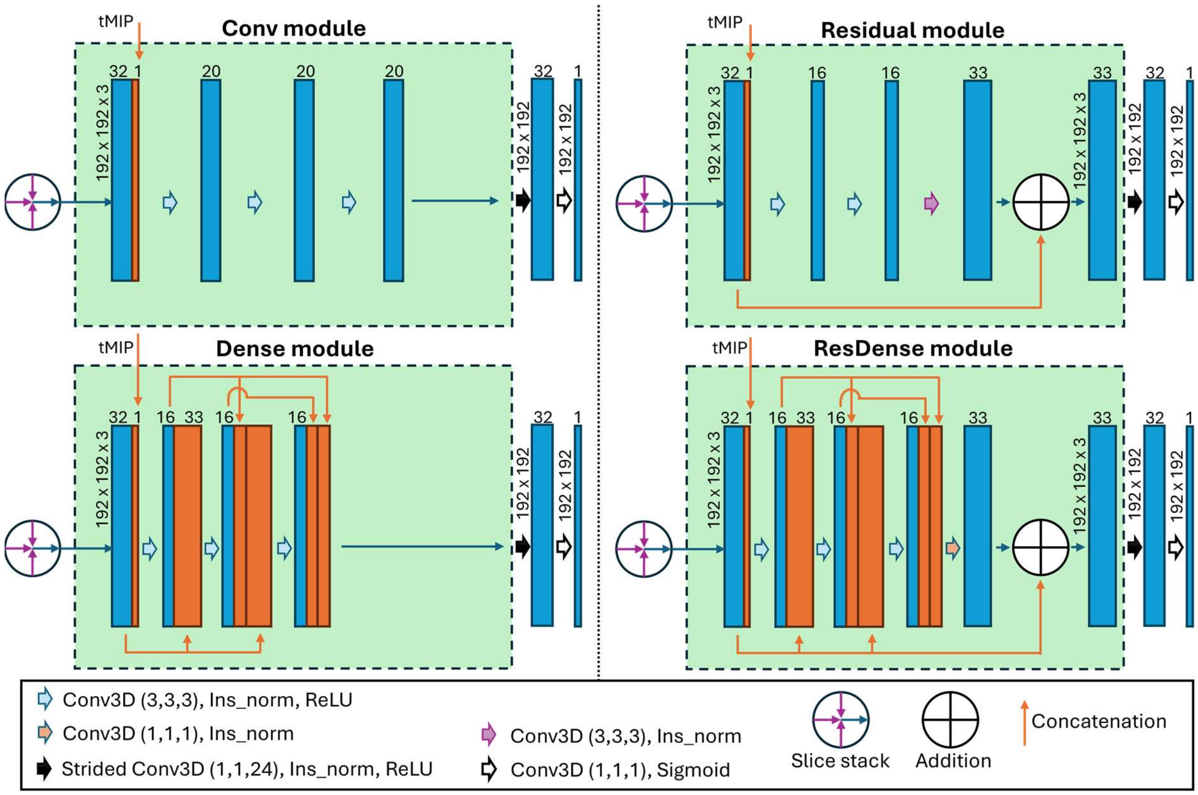

Figure S3: Tested refinement modules for 4DST. The last 2 layers, strided convolution and convolution with sigmoid activation are present in all modules. Convolution kernel sizes are shown in the legend.

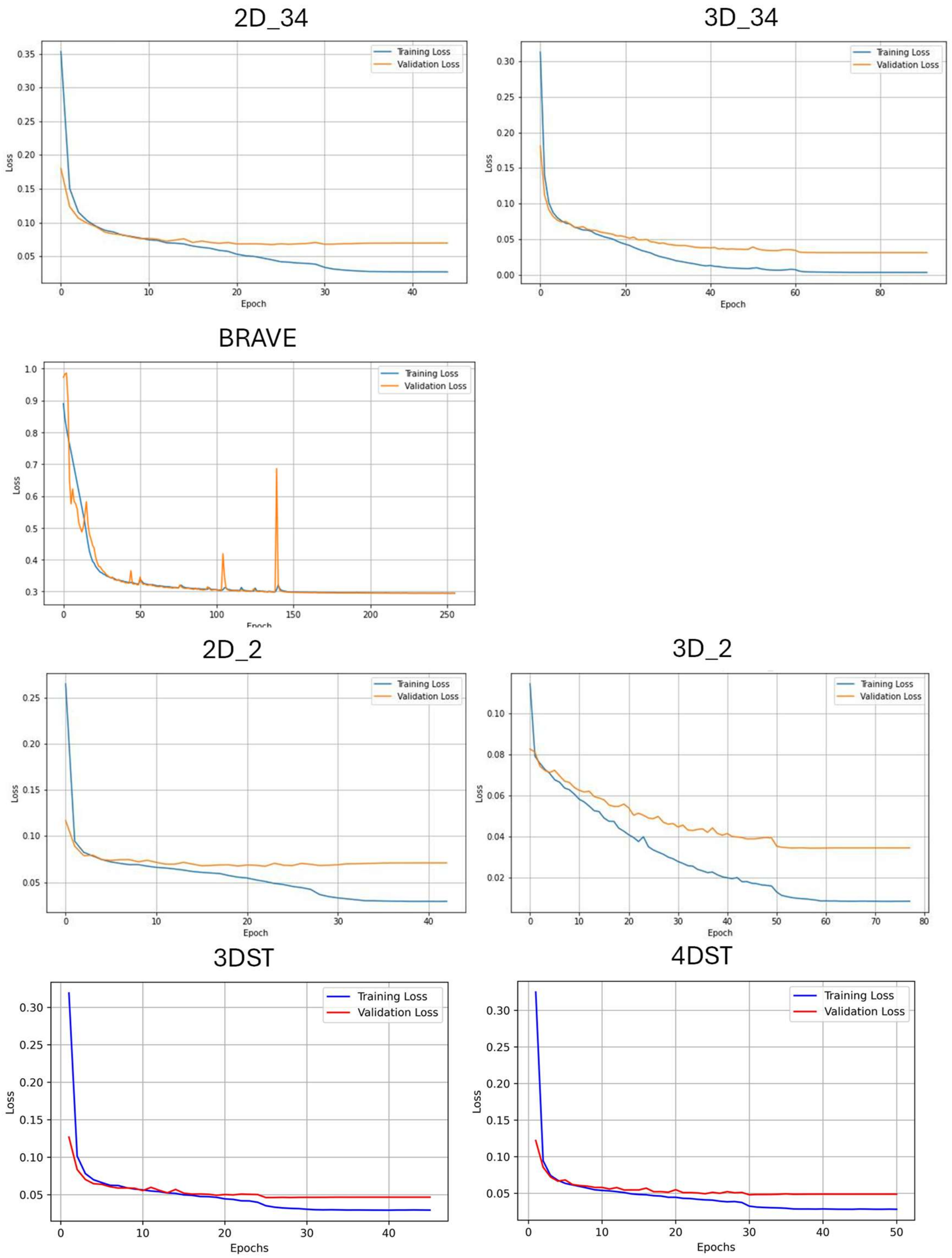

Figure S4: Training curves of compared models, 2D_34 and 3d_34 correspond to models trained with batch size = 34, 2D_2 and 3D_2 correspond to models trained with batch size = 2. BRAVE-Net was trained with batch size = 34. 3DST and 4DST were trained with batch size = 2.

Table S1: 3DST ablation, kernel sizes and pre-normalization layer (instance normalization) (mean ± std.dev)

Kernel sizes	Prenorm	DSC	clDice	HD	sensitivity	specificity	accuracy	precision
3,3,3,3,3,3,3	×	0.856±0.044	0.842±0.035	7.3±1.2	0.842±0.090	0.99983±0.00005	0.9982±0.0008	0.981±0.006
3,3,5,7,5,3,3	×	<u>0.860±0.037</u>	<u>0.851±0.031</u>	7.1±1.1	0.842±0.085	<u>0.99984±0.00004</u>	0.9982±0.0008	<u>0.983±0.003</u>
5,5,5,5,5,5,5	×	0.858±0.029	0.847±0.025	7.1±1.3	<u>0.846±0.061</u>	0.99983±0.00003	<u>0.9982±0.0006</u>	0.980±0.004
9,7,5,3,5,7,9	×	0.855±0.042	0.844±0.034	<u>7.1±1.3</u>	0.840±0.083	0.99982±0.00006	0.9982±0.0007	0.980±0.007
3,3,5,7,5,3,3	✓	**0.867±0.034**	**0.856±0.028**	**6.4±0.9**	**0.852±0.07**	**0.99986±0.00003**	**0.9982±0.0007**	**0.984±0.005**

Kernel sizes indicate the convolutional kernel sizes in the time dimension per encod/decod stages (no difference between conv layers in the same stage). Best indicated with bold, second best underlined

Table S2: External validation DSC metrics from AVM data set (whole volume and cropped AVM lesion), 10 and 40 spoke 4DMRA test-set (50 and 200 ms temporal resolution respectively), and slice downsampled test-set (mean ± std.dev)

(A) AVM Whole Volume

Model	DSC	clDice	HD	sensitivity	specificity	accuracy	precision
IsoF	0.680±0.038	0.565±0.053	12.7±2.0	0.672±0.045	0.99946±0.00024	0.9962±0.0008	0.921±0.043
2D_34	0.800±0.013	0.780±0.014	10.1±1.8	0.801±0.032	<u>0.99984±0.00005</u>	0.9978±0.0005	<u>0.981±0.003</u>
3D_34	0.698±0.066	0.722±0.033	15.0±4.5	0.677±0.102	0.99973±0.00012	0.9963±0.0015	0.962±0.011
BRAVE	0.672±0.082	0.730±0.033	15.0±5.0	0.605±0.091	0.99976±0.00025	0.9958±0.0017	0.963±0.035
2D_2	<u>0.808±0.005</u>	<u>0.793±0.009</u>	<u>9.4±1.4</u>	<u>0.829±0.023</u>	**0.99984±0.00006**	<u>0.9981±0.0004</u>	**0.982±0.004**
3D_2	0.707±0.060	0.727±0.036	14.8±4.7	0.694±0.090	0.99975±0.00009	0.9965±0.0014	0.967±0.006
3DST	**0.809±0.008**	**0.798±0.010**	**9.4±1.1**	0.801±0.029	0.99983±0.00002	0.9979±0.0005	0.978±0.007
4DST	0.792±0.008	0.790±0.011	10.1±1.6	**0.871±0.038**	0.99961±0.00011	**0.9982±0.0005**	0.962±0.005

(B) Cropped AVM lesion

Model	DSC	clDice	HD	sensitivity	specificity	accuracy	precision
IsoF	0.757±0.064	0.649±0.103	9.7±4.0	0.782±0.102	0.99762±0.00192	0.9788±0.0121	0.911±0.117
2D_34	0.850±0.029	0.824±0.054	7.8±3.7	0.821±0.038	**0.99979±0.00009**	0.9876±0.0067	<u>0.990±0.014</u>
3D_34	0.630±0.093	0.638±0.082	12.6±6.7	0.545±0.145	0.99937±0.00053	0.9632±0.0215	0.955±0.062
BRAVE	0.531±0.127	0.584±0.088	12.9±7.2	0.410±0.131	0.99752±0.00367	0.9539±0.0268	0.898±0.105
2D_2	0.846±0.032	0.825±0.068	<u>7.4±3.1</u>	0.831±0.037	<u>0.99970±0.00022</u>	0.9884±0.0055	0.982±0.027
3D_2	0.649±0.086	0.634±0.068	12.5±6.7	0.565±0.125	0.99938±0.00026	0.9649±0.0203	0.964±0.045
3DST	**0.867±0.017**	**0.852±0.028**	**6.8±2.9**	<u>0.841±0.041</u>	0.99970±0.00019	<u>0.9893±0.0056</u>	**0.992±0.008**
4DST	<u>0.852±0.016</u>	<u>0.828±0.069</u>	7.6±3.3	**0.902±0.040**	0.99924±0.00028	**0.9915±0.0055**	0.980±0.022

(C) 10-spoke reconstructed (50 ms temporal resolution)

Model	DSC	clDice	HD	sensitivity	specificity	accuracy	precision
IsoF	0.733±0.052	0.714±0.072	10.2±1.8	0.782±0.059	0.99902±0.00079	0.9965±0.0011	0.899±0.086
2D_34	0.836±0.021	0.832±0.018	7.4±1.1	0.836±0.052	0.99984±0.00006	0.9980±0.0006	0.983±0.005
3D_34	0.818±0.023	0.822±0.018	7.5±1.1	0.817±0.054	0.99982±0.00005	0.9978±0.0006	0.981±0.004
BRAVE	0.822±0.033	0.826±0.027	7.6±1.2	0.816±0.077	0.99978±0.00011	0.9977±0.0008	0.978±0.009
2D_2	0.843±0.020	0.844±0.020	7.5±1.1	**0.866±0.058**	0.99986±0.00005	<u>0.9983±0.0006</u>	0.986±0.003
3D_2	0.816±0.028	0.823±0.020	7.7±1.3	0.822±0.067	0.99981±0.00006	0.9978±0.0007	0.981±0.004
3DST	<u>0.864±0.029</u>	<u>0.851±0.027</u>	<u>6.5±0.8</u>	0.851±0.063	<u>0.99988±0.00005</u>	0.9982±0.0007	<u>0.988±0.003</u>
4DST	**0.868±0.027**	**0.856±0.022**	**6.4±0.9**	<u>0.862±0.041</u>	**0.99988±0.00005**	**0.9984±0.0005**	**0.988±0.003**

best performing indicated in bold, second best underlined

Table S2 continued

(D) 40-spoke reconstructed (200 ms temporal resolution)							
Model	DSC	clDice	HD	sensitivity	specificity	accuracy	precision
IsoF	0.729±0.036	0.715±0.060	10.2±2.1	0.794±0.042	0.99900±0.00063	0.9966±0.0007	0.898±0.069
2D_34	0.836±0.020	0.835±0.016	7.1±0.9	0.835±0.053	0.99978±0.00009	0.9979±0.0007	0.975±0.012
3D_34	0.819±0.020	0.827±0.014	7.4±0.9	0.831±0.050	0.99971±0.00013	0.9978±0.0005	0.971±0.012
BRAVE	0.819±0.031	0.831±0.024	8.1±1.3	0.835±0.075	0.99962±0.00021	0.9977±0.0008	0.964±0.017
2D_2	0.846±0.023	0.847±0.020	7.2±0.9	**0.867±0.060**	**0.99981±0.00006**	**0.9983±0.0006**	**0.980±0.009**
3D_2	0.816±0.028	0.824±0.019	7.7±1.3	0.833±0.067	0.99969±0.00014	0.9978±0.0006	0.969±0.010
3DST	0.850±0.021	0.845±0.020	6.7±0.9	0.842±0.058	0.99979±0.00011	0.9980±0.0007	0.978±0.010
4DST	**0.856±0.024**	**0.854±0.019**	**6.6±0.8**	0.856±0.047	0.99976±0.00008	0.9981±0.0006	0.974±0.010

(E) Slice downsampled							
Model	DSC	clDice	HD	sensitivity	specificity	accuracy	precision
IsoF	0.717±0.038	0.692±0.070	6.0±0.9	0.732±0.073	0.99929±0.00053	0.9964±0.0008	0.915±0.066
2D_34	0.844±0.021	0.835±0.020	4.6±0.7	0.845±0.049	0.99982±0.00007	0.9982±0.0005	0.980±0.008
3D_34	0.819±0.023	0.816±0.023	5.0±0.6	0.854±0.052	0.99971±0.00009	0.9981±0.0005	0.970±0.009
BRAVE	0.824±0.028	0.824±0.024	5.0±0.7	0.850±0.076	0.99966±0.00018	0.9980±0.0006	0.965±0.016
2D_2	0.848±0.020	0.844±0.021	4.7±0.7	**0.872±0.059**	0.99982±0.00005	0.9985±0.0005	0.981±0.007
3D_2	0.817±0.022	0.815±0.023	5.1±0.5	0.859±0.061	0.99973±0.00012	0.9982±0.0005	0.973±0.012
3DST	0.869±0.038	0.854±0.032	4.2±0.8	0.852±0.072	0.99984±0.00005	0.9983±0.0006	0.981±0.007
4DST	**0.874±0.031**	**0.857±0.030**	**4.1±0.6**	0.865±0.047	**0.99986±0.00005**	**0.9985±0.0005**	**0.984±0.007**

best performing indicated in bold, second best underlined
